# Prevalence of Prefrailty and Frailty Among Older Adults in Germany: A Systematic Review, Meta-Analysis and Meta-Regression

**DOI:** 10.3389/fmed.2022.870714

**Published:** 2022-04-22

**Authors:** André Hajek, Benedikt Kretzler, Hans-Helmut König

**Affiliations:** Department of Health Economics and Health Services Research, University Medical Center Hamburg-Eppendorf, Hamburg Center for Health Economics, Hamburg, Germany

**Keywords:** frailty, Germany, prevalence, old age, systematic review, aged 80 and over

## Abstract

**Background:**

Various studies have identified the prevalence of prefrailty and frailty among older adults in Germany. Nevertheless, there is no review systematically synthesizing these studies. Thus, our aim was to close this gap in knowledge. Moreover, another aim was to perform a meta-analysis to synthesize the pooled prevalence of prefrailty and frailty. A further aim was to explore potential sources of heterogeneity based on a meta-regression.

**Methods:**

A number of three electronic databases (PubMed, PsycINFO, and CINAHL) were searched (plus an additional hand search). The observational studies that determine the prevalence of frailty among older adults aged 65 years and above in Germany were included, whereas disease-specific samples were excluded. Data extraction included the description of the sample, operationalization of frailty, statistical analysis, sample characteristics and main findings. The established Joanna Briggs Institute (JBI) standardized critical appraisal instrument for prevalence studies was used for evaluating the quality of the studies. Important steps were performed by two reviewers.

**Results:**

In sum, a number of 12 studies were included. The prevalence of frailty varied from about 2.4 to 25.6%. The pooled prevalence of frailty was 13.7% (95% CI: 9.0 to 18.5%). There was a significant heterogeneity among the studies (*I*^2^ = 98.9%, *p* < 0.001). The pooled prevalence of prefrailty was 40.2% (95% CI: 28.3 to 52.1%; *I*^2^ = 99.6%, *p* < 0.001). Some evidence of a publication bias exists. Meta-regressions showed that some of the heterogeneity was explained by the tool to quantify frailty and the average age of the respective sample.

**Conclusion:**

Particularly, the high prevalence of prefrailty should be highlighted since it is important to prevent individuals in old age from developing to frail status. This knowledge is important for the German society as a whole and for relevant stakeholders.

**Systematic Review Registration:**

PROSPERO, identifier: CRD42021293648.

## Introduction

Common attributes of frailty are a lack of physiological reserve and an increased vulnerability to stressors (1). Clegg et al. (2) defined it as “a state of vulnerability to poor resolution of homoeostasis after a stressor event” (p. 759).

Former research has demonstrated that frailty can increase the likelihood of institutionalization (3) and mortality (4, 5). Additionally, frailty can contribute to high economic costs (6). Against the backdrop of demographic aging, it is often assumed that the number of individuals with frailty will rise considerably (7). This underlines the importance of knowledge about the general prevalence of frailty.

For example, a recent systematic review and meta-analysis showed a pooled prevalence of frailty of 7.4% (95% confidence interval: 6.1–9.0%) among Japanese community-dwelling older people (8). While some studies also exist identifying the prevalence of prefrailty and frailty among older adults in Germany [e.g., among individuals aged 65 years and above: 2.8% were frail (9); among individuals aged 85 years and over: 31.7% of individuals were frail (10)]; a systematic review is lacking which systematically synthesizes the current evidence.

Thus, our first aim was to systematically summarize this evidence among older adults (i.e., 65 years and older) in Germany. Our second aim was to perform a meta-analysis to synthesize the pooled prevalence of frailty and also prefrailty among older adults in Germany—which can help to obtain more accurate prevalence rates of frailty. This is the important basic information for individuals involved in frailty research. Third, a meta-regression will be conducted to identify the impact of potentially moderating factors (such as tools used to quantify frailty). Our work may also help to identify knowledge gaps and can consequently inspire upcoming frailty research.

## Methods

Our current work is in accordance with the Preferred Reporting Items for Systematic Reviews and Meta-Analysis guidelines (11). Additionally, it has been registered in the International Prospective Register of Systematic Reviews (PROSPERO: CRD42021293648).

In December 2021, three electronic databases (MEDLINE, PsycINFO, and CINAHL) were searched. Our search strategy (MEDLINE) is displayed in Table 1.

**Table 1:** MEDLINE search algorithm.

#1	Frail*
#2	Frailty syndrome [MeSH Terms]
#3	#1 OR #2
#4	German*
#5	Germany [MeSH Terms]
#6	#4 OR #5
#7	#3 AND #6

Using two steps (title/abstract screening first and after that full-text screening), two reviewers (AH and BK) assessed the suitability. In addition, a hand search of the reference lists of retrieved papers was conducted. The discussions were used to resolve any discrepancies. This practice was also applied when there were any discrepancies in extracting the data or assessment of the study quality.

Main inclusion criteria for the screening included (i) cross-sectional and longitudinal observational studies identifying the prevalence of frailty among older adults (65 years and over) residing in Germany, (ii) studies adequately assessing frailty, (iii) studies published in peer-reviewed journals, (iv) and studies published in German or English language.

The cutoff for older adults (i.e., 65 years) was selected because in Germany, the age of 65 years was set in past years for retirement and commonly characterizes the transition from middle age to old age.

Main exclusion criteria included (i) the studies solely investigating samples with a specific disorder (e.g., individuals with mental disorders), (ii) the assessment of key variables (i.e., frailty) not appropriate (e.g., single item with two values to quantify frailty), and (iii) the studies not published in peer-reviewed journal.

Disease-specific samples were excluded since they may not be generalizable to the general population in late life.

No restrictions were applied with regard to the time and location of publication (except for Germany).

Prior to final eligibility criteria, a pretest was done (i.e., both reviewers screened a sample of 100 titles/abstracts and discussed their results). However, eligibility criteria were not refined.

With regard to the data extraction, while one reviewer (BK) extracted the data, a second reviewer (AH) checked the data extraction. The data extraction focused on the description of the sample, operationalization of frailty, statistical analysis, sample characteristics, and main findings.

To assess the quality of the studies, we used the established Joanna Briggs Institute (JBI) standardized critical appraisal instrument for prevalence studies (12). The resulting score ranged from 0 to 9 (higher values indicate higher study quality and less risk of bias).

Regarding meta-analysis, random-effect models were used to pool proportions across the studies. The underlying assumption of the random-effect models is that heterogeneity across studies exists. Following the recommendations, heterogeneity between studies was assessed using the I^2^ statistic [I^2^ values from 25 to 50%: low; 50 to 75%: moderate; 75% or more: high heterogeneity (13)]. The established “metaprop” (14) command was used to conduct the meta-analysis.

Regarding meta-regression (including these factors: mean age, assessment of frailty, and risk of bias score), the “meta regress” command from Stata 16 was used [more precisely, random-effects, with restricted maximum likelihood; moreover, Knapp-Hartung adjustment for the standard errors was used (15)]. The effect sizes were recalculated in a first step (16) because the coefficients are initially scaled as double arcsin values rather than proportions. Such meta-regressions were computed to examine the roots of heterogeneity (17).

A funnel plot and the Egger's test (*p* < 0.05 indicates publication bias) were applied to examine the publication bias. Stata 16.1 (College Station, TX, USA) was used for all analyses.

## Results

### Overview: Included Studies

**Figure 1:**
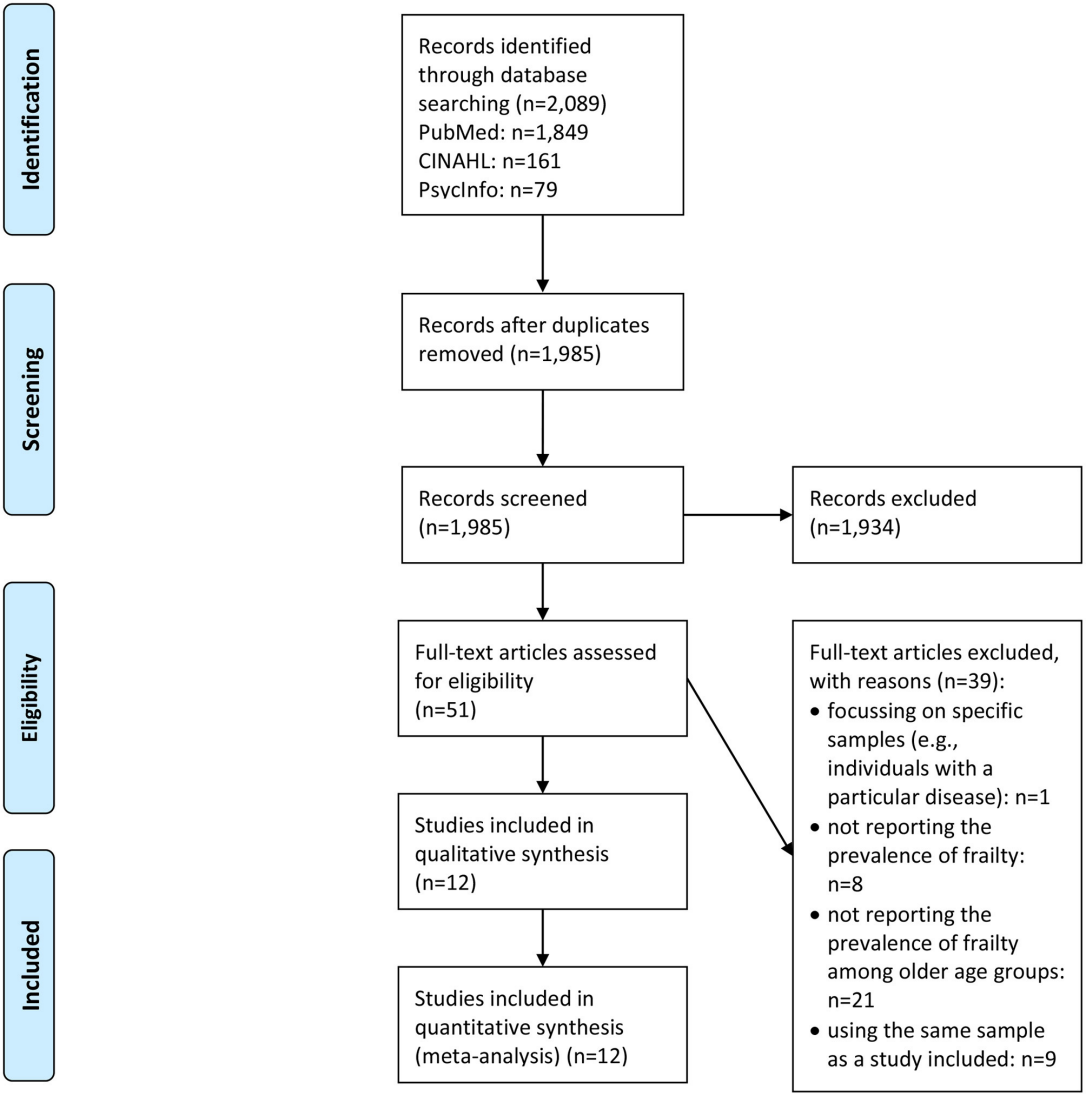
PRISMA flow diagram.

In Figure 1 (11), the selection process is displayed. In sum, a number of 12 studies were included in our systematic review—and also in meta-analysis. In Table 2, a study overview that includes the main findings is given (10, 18, 20, 21, 23–30).

**Table 2:** Study overview and key findings.

**References**	**Assessment of frailty**	**Study type**	**Sample descriptions**	**Sample size**	**Age females in total sample**	**Living situation (community-dwelling/ institutionalized)**	**Results: Prevalence of frailty**
Bollwein et al. (18)	According to Fried et al. (19) at least three out of five criteria: - weight loss (>4.5 kg in the last year) - exhaustion (self-reported feeling that everything was an effort or that one could not get “going” more than 2 times a week) - low grip strength (Jamar dynamometer, men ≤ 29–32 kg, women ≤ 17–21 kg stratified by BMI) - low walking speed (depending on gender and height > 6–7 s/4.57 m) - low physical activity (Minnesota Leisure Time Activities Questionnaire) (men <383 kcal/ week, women <270 kcal/week)	Cross-sectional	Individuals without cognitive impairment	*n =* 206	Median: 76 75–96 Female: 66.0%	Community-dwelling	15.5%
Braun et al. (20)	- Physical Frailty Phenotype according to Fried et al. (19)	Cross-sectional	Community-dwelling individuals receiving physiotherapy treatment in an outpatient practice	*n =* 96	M: 73 SD: 6 65–87 Females: 63%	Community-dwelling	Fried: 4%
Braun et al. (21)	According to Fried et al. (19) at least three out of five criteria: - grip strength: assessment protocol proposed by Roberts et al. (22) - gait speed: over 4.57 m - physical activity: Minnesota Leisure Time Activity Questionnaire - weight loss: >4.5 kg unintentionally within the prior year - exhaustion: Centre for Epidemiological Studies—Depression Scale	Cross-sectional	Individuals seeking outpatient physiotherapy	*n =* 258	M: 73.8 SD: 5.6 ≥ 65 Females: 62.0%	Community-dwelling	17.8% (95% CI: 13.2%-22.5%)
Castell et al. (23)	According to Fried et al. (19) at least three out of five criteria: - unintentional weight loss of ≥5% in the last year - low energy: Centre for Epidemiologic Studies Depression Scale	Longitudinal (baseline in 2009/2010, follow-ups not specified)	European Project on OSteoArthritis	*n =* 336	M: 74.0 SD: 5.0 65–85 Females: 50.8%	Community-dwelling	5.6% (95% CI: 3.1%-8.1%)
	- weakness: grip strength adjusted for body mass index - slowness: three meters, adjusted for sex and height - low physical activity: lowest quintile						
Dallmeier et al. (24)	32 items regarding activities of daily life, instrumental activities of daily life, multimorbidities, psychosocial anamnesis, self-perception, risk of fall, and functional measurements	Longitudinal (baseline in 2009/2010, follow-ups not specified)	Active and Function in the Elderly in Ulm	*n =* 1,204	M: 74.0 IQR: 70.1–81.1 ≥ 65 Females: 57.5%	Community-dwelling	21.7%
Dapp et al. (25)	LUCAS Functional Ability Index	Longitudinal (2007–2017, six waves)	Longitudinal Urban Cohort Ageing Study	*n =* 2,012	2007: M: 76.2 SD: 6.5 ≥ 66 2017: M: 82.8 SD: 4.6 ≥ 76 Females: 2007: 63.1% 2017: 61.6%	Community-dwelling	2007: 25.6% 2017: 36.3%
Du et al. (26)	Having 3 and more of the following criteria: Exhaustion, low weight, low physical activity, low walking speed and low grip strength	Cross-sectional	German Health Interview and Examination Survey for Adults	*n =* 1,833	65–69: 34.8% 70–74: 42.8% 75–79: 22.3% Female: 54.0%	Community-dwelling	2.5%
Haider et al. (27)	According to the SHARE frailty index: - exhaustion - weight loss - weakness - slowness - low activity	cross-sectional	European Health Interview Survey	*n =* 2,457	≥ 65 Females: not reported	Community-dwelling	8.2%
Hajek et al. (10)	Canadian Study of Health and Aging Clinical Frailty Scale	Longitudinal (FU wave 7 to FU wave 9)	Needs, Health Service Use, Costs and Health-Related Quality of Life in a Large Sample of Oldest-Old Primary Care Patients	*n =* 510	M: 90.3 SD: 2.7 ≥85 Females: 68.0%	Not being institutionalized: 88.0% being institutionalized: 12.0%	12.8%
Saum et al. (28)	Frailty Index (34 items concerning diseases, general health, difficulties in activities of daily life and instrumental activities of daily life, and symptoms)	Longitudinal (baseline in 2000/2002, follow-ups not specified)	“Epidemiologische Studie zu Chancen der Verhütung, Früherkennung und optimierten Therapie chronischer Erkrankungen in der älteren Bevölkerung”	*n =* 3,810	M: 62.0 65–69: 59.4% 70–75: 40.6% Female: 54.6%	Community-dwelling	13.8%
Stephan et al. (29)	KORA-Age Frailty Index (30 items covering 10 diseases, 13 measures of functioning and seven signs and symptoms)	Longitudinal (2009–2012, two waves)	Cooperative Health Research in the Region of Augsburg-Age Study	*n =* 1,076	M: 76 65–70: 21.2% 70–74: 21.3% 75–79: 22.6% 80–84: 22.4% >84: 1.6 Females: 50.1%	Community-dwelling	18.4%
Zimmermann et al. (30)	According to Fried et al. (19) at least three out of five criteria: - exhaustion: strongly noticing that one has less energy with increasing age or that one increasingly has to restrict one's activities with one's increasing age - unintentional weight loss: having unintentionally lost a considerable amount of weight in the last 12 months - weakness: measured with a hand-held dynamometer - grip strength: being in the lowest quartile of the overall sample - physical activity: no or low physical activity, assessed through a list of predefined activities	Cross-sectional	Quality of Life and Well-Being of the Very Old in North Rhine-Westphalia	*n =* 1,577	M: 84.9 SD: 4.0 ≥ 80 Females: 63.2%	Private household: 89.3% nursing home: 10.7%	18.7%

A total of six studies were longitudinal (where we used the prevalence at baseline for meta-analysis) (10, 23–25, 28), whereas the other six studies were cross-sectional. Data were mainly used from the well-known German studies (e.g., “German Health Interview and Examination Survey for Adults” or “Quality of Life and Well-Being of the Very Old in North Rhine-Westphalia”). The sample size ranged from 96 (20) to 3,810 individuals (28). All the studies were published in the past 10 years (year 2013 onward).

Different tools were used to assess frailty [e.g., CSHA CFS (31) or according to the study of Fried et al. (19)]. The proportion of women ranged from 50 to 68%. Most samples had an average age of about 70 to 80 years. A number of ten studies solely used data from individuals residing in private households. More details are shown in Table 2.

### Meta-Analysis and Meta-Regression

**Figure 2:**
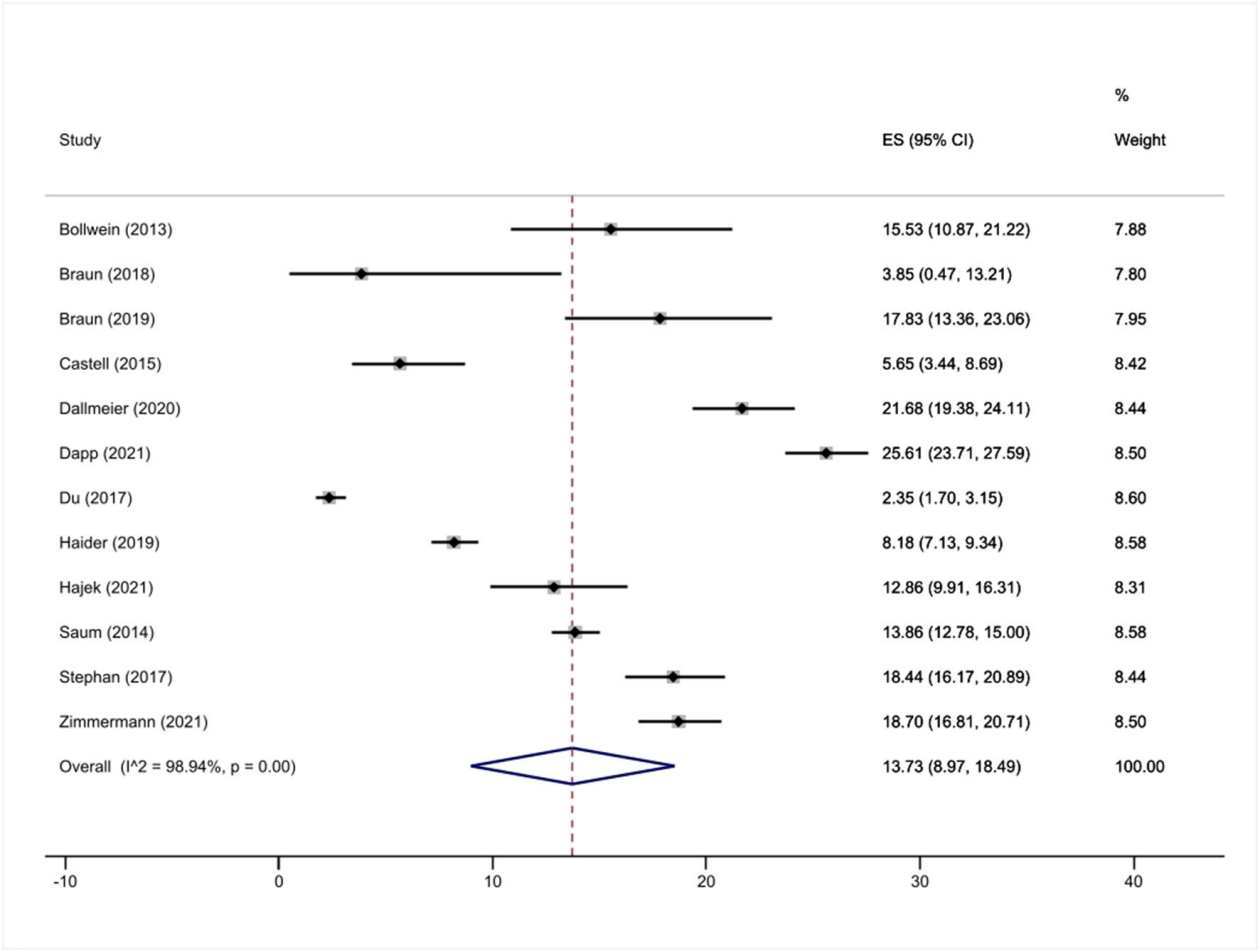
Meta-analysis (frailty).

In total, the estimated overall prevalence of frailty was 13.7% (95% CI: 9.0 to 18.5%, Figure 2; ranging from 3.9 to 25.6%). There was a significant heterogeneity between the studies (*I*^2^ = 98.9%, *p* <0.001). The pooled prevalence of frailty was 15.9% (95% CI: 11.8 to 20.0%, ranging from 12.9 to 18.7%; *I*^2^ = 80.5%) when we only included samples with an average age of at least 80 years, whereas the pooled prevalence of frailty was 13.1% (95% CI: 7.5 to 18.6%, ranging from 3.9 to 25.6%; *I*^2^ = 99.1%) when we only included samples with an average age of below 80 years.

**Figure 3:**
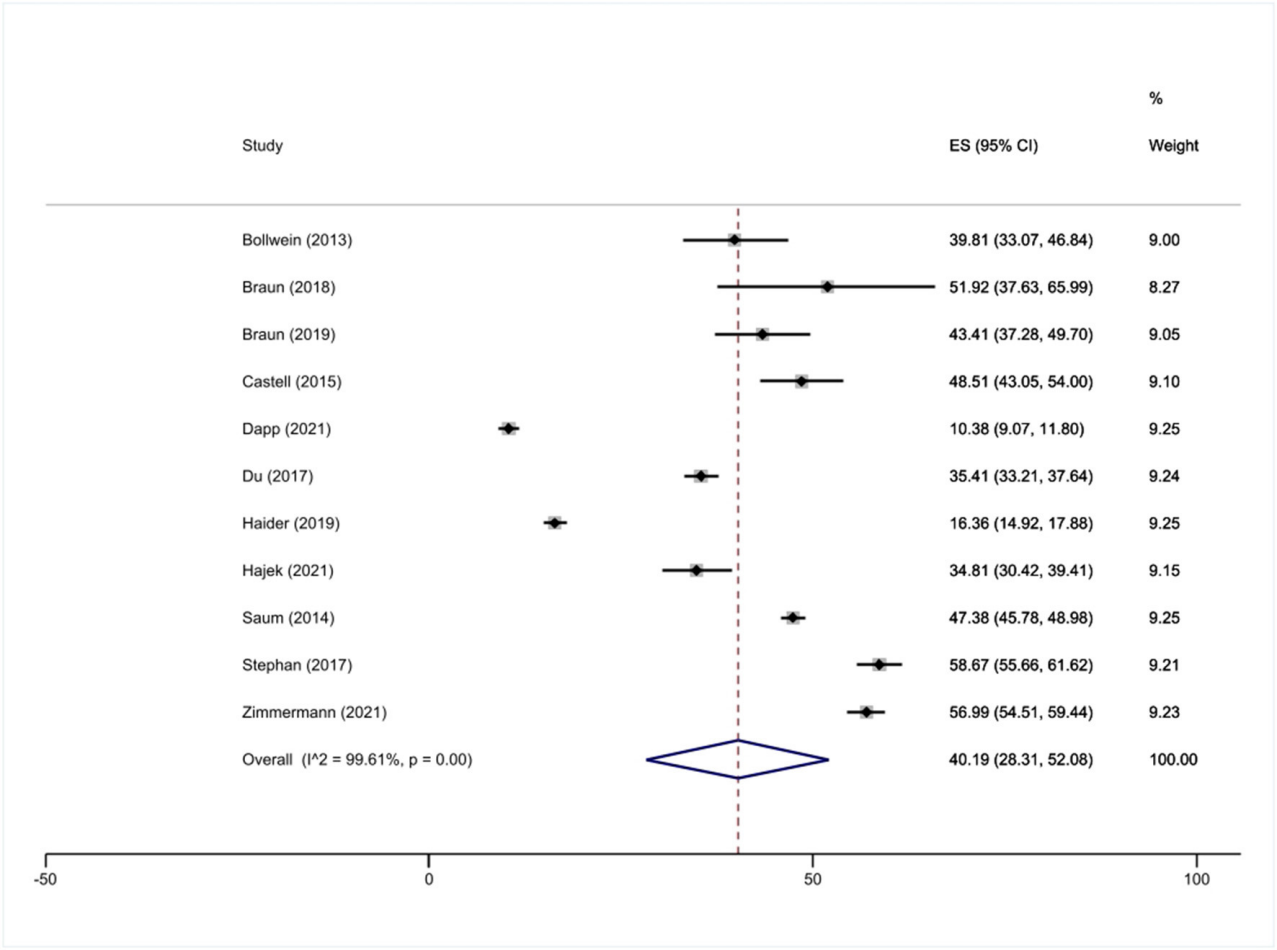
Meta-analysis (prefrailty).

Furthermore, the pooled prevalence of prefrailty was 40.2% (95% CI: 28.3 to 52.1%; ranging from 10.4 to 58.7%; *I*^2^ = 99.6%, *p* <0.001, Figure 3). The pooled prevalence of prefrailty was 44.0% (95% CI: 28.0 to 60.0%, ranging from 34.8 to 57.0%; *I*^2^ = 97.7%) when we only included samples with an average age of at least 80 years, whereas the pooled prevalence of frailty was 38.8% (95% CI: 25.2 to 52.4%, ranging from 10.4 to 58.7%; *I*^2^ = 99.7%) when we only included samples with an average age of below 80 years.

**Figure 4:**
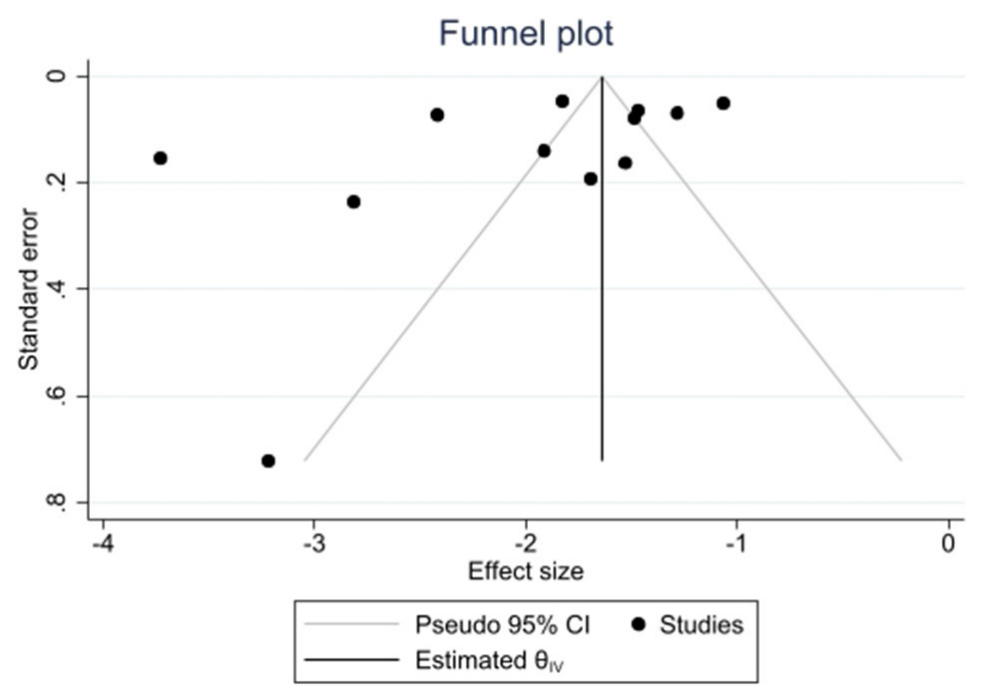
Funnel plot.

Moreover, a meta-regression revealed that frailty prevalence was dependent of the average age in the sample and the assessment of frailty, whereas the risk of bias score did not achieve statistical significance (Table 3). The proportion of variance explained by these factors was 50.9%. The funnel plot (Figure 4) and the Egger's test (*p* = 0.06) partly suggested asymmetry of data—which implies a publication bias.

**Table 3:** Meta-regression analysis of factors affecting heterogeneity (prevalence of frailty).

**Variable**	**Coefficient (95% confidence interval)**	***p*-value**
Assessment: - CSHA-CFS [Reference: Fried et al. (19)]	−0.32 (−2.13 to 1.49)	0.69
- Frailty Index	1.45 (0.51 to 2.39)	0.01
Average age	0.08 (0.004 to 0.15)	0.042
Risk of bias score	−0.14 (−0.41 to 0.12	0.23

### Quality Assessment or Risk of Bias Assessment

In Table 4, the risk of bias assessment or quality assessment is shown. In total, the scores varied from 4 to 9, with an average score of 7.3 (SD: 1.9). This reflects that the quality was rather high and the risk of bias was consequently quite small. Most often, limitations were the missing or inappropriate response rate (*n* = 8) and the missing or insufficient description or discussion of the model assumptions in these studies (*n* = 5). However, it should be acknowledged that some of these studies focused on reporting the prevalence and thus did not focus on the determinants of frailty in late life (and complex analytical models).

**Table 4:** Quality assessment/risk of bias assessment.

	**Quality scores (from 1 to 9)**									
	**Items**									**Quality score (from 1 to 9; higher scores indicate less risk of bias)**
**Study**	**1**	**2**	**3**	**4**	**5**	**6**	**7**	**8**	**9**	**Total**
Bollwein et al. (18)	N	N	N	Y	Y	Y	Y	N	N	4
Braun et al. (20)	N	N	N	Y	Y	Y	Y	N	N	4
Braun et al. (21)	N	Y	N	Y	Y	Y	Y	N	N	5
Castell et al. (23)	Y	Y	Y	Y	Y	Y	Y	N	Y	8
Dallmeier et al. (24)	Y	Y	Y	Y	Y	Y	Y	Y	N	8
Dapp et al. (25)	Y	Y	Y	Y	Y	Y	Y	Y	N	8
Du et al. (26)	Y	Y	Y	Y	Y	Y	Y	Y	Y	9
Haider et al. (27)	Y	Y	Y	Y	Y	Y	Y	N	N	7
Hajek et al. (6)	Y	Y	Y	Y	Y	Y	Y	Y	Y	9
Saum et al. (28)	Y	Y	Y	Y	Y	Y	Y	Y	N	8
Stephan et al. (29)	Y	Y	Y	Y	Y	Y	Y	Y	N	8
Zimmermann et al. (30)	Y	Y	Y	Y	Y	Y	Y	Y	Y	9

*The Joanna Briggs Institute (JBI) standardized critical appraisal instrument for prevalence studies was used. Y, Yes; N, No; U, Unclear; 1: Was the sample frame appropriate to address the target population? 2: Were study participants sampled in an appropriate way? 3: Was the sample size adequate? 4: Were the study subjects and the setting described in detail? 5: Was the data analysis conducted with sufficient coverage of the identified sample? 6: Were valid methods used for the identification of the condition? 7: Was the condition measured in a standard, reliable way for all participants? 8: Was there appropriate statistical analysis? 9: Was the response rate adequate, and if not, was the low response rate managed appropriately?*

## Discussion

### Main Findings

Our aim was to determine the prevalence of frailty among older adults living in Germany. Another aim was to identify the potential sources of heterogeneity using a meta-regression.

The pooled prevalence of frailty was 13.7% (95% CI: 9.0 to 18.5%) and the pooled prevalence of prefrailty was 40.2% (95% CI: 28.3 to 52.1%). Considerable heterogeneity among the studies was determined. Some evidence of a publication bias exists. However, we think that more plausible explanations for the lack of publications could be that studies only had a small sample size or used data from convenience samples. Meta-regression showed that some of the heterogeneity can be explained by the tool to quantify the frailty and the average age. This appears very plausible. For example, Saum et al. showed that the prevalence of frailty was 4.5% among individuals aged 50 to 54 years, whereas the prevalence was 17.0% among individuals aged 70 to 75 years. Additionally, the prevalence of prefrailty was 32.5% among individuals aged 50 to 54 years, whereas the prevalence was 49.4% among individuals aged 70 to 75 years.

### Comparability of the Included Studies

Substantial heterogeneity among the studies was identified. For example, the considerable higher prevalence rates of frailty were identified when the Frailty Index was used [compared to Fried et al. (19)]. It should be emphasized that no consensus exists on a single instrument for quantifying frailty (20).

It should be noted that a few studies exclusively used data from oldest old individuals (10, 30). This is worth noting because the prevalence of frailty commonly increases with age (8)—as noted above. Furthermore, while two studies also included individuals living in institutionalized settings (10, 30), the remaining studies explicitly examined community-dwelling individuals.

### Study Quality

In total, a quite high quality of the studies included in our work was identified. For example, most of the studies used data from well-conducted samples. Frequent shortcomings were that the response rate was not clearly displayed or that the underlying assumptions of the analytical choice were not clearly described.

### Gaps in Knowledge and Guidance for Future Studies

Our work identified some gaps in knowledge: First, far more research is required based on the samples which also include individuals residing in institutionalized settings. Moreover, more studies that examine the prevalence of frailty based on the representative samples and comparable assessments is required.

Beyond that, more longitudinal studies are required to determine the factors that lead to frailty in older adults in Germany. Additionally, since the existing longitudinal studies are mainly restricted in time span, more population-based longitudinal studies are required examining individuals over several *decades* (e.g., from middle age to highest age). These gaps may inspire future frailty research.

### Strengths and Limitations

Our current work has some strengths and limitations. This is the first systematic review synthesizing the prevalence of frailty among older adults in Germany. Important procedures were done independently by two reviewers. An additional hand search was performed. Furthermore, a meta-analysis was conducted which can result in more accurate prevalence rates of frailty (when compared to individual empirical studies). Moreover, a meta-regression was done which can assist to clarify the influence of moderating factors. Due to the exclusion of non-peer-reviewed articles, some appropriate studies (e.g., gray literature) might be excluded. However, this choice was done to ensure a certain quality of the studies included in our work.

## Conclusion

The pooled prevalence of frailty was 13.7% (95% CI: 9.0 to 18.5%) and the pooled prevalence of prefrailty was 40.2% (95% CI: 28.3 to 52.1%). Particularly, the high prevalence of prefrailty should be highlighted since it is important to prevent individuals in old age from developing to frail status. This knowledge is important for the German society as a whole and for relevant stakeholders. More longitudinal studies are required to reveal the factors contributing to frailty.

## Data Availability Statement

The original contributions presented in the study are included in the article/supplementary material, further inquiries can be directed to the corresponding author.

## Author Contributions

The study concept was developed by AH, BK, and H-HK. The manuscript was drafted by AH and critically revised by BK and H-HK. The search strategy was developed by AH and H-HK. The study selection, data extraction, and quality assessment were performed by AH and BK, with H-HK as a third party in case of disagreements (i.e., if clarification was still needed after discussion between AH and BK). Meta-analysis and meta-regressions were performed by AH, with critical assessment by BK and H-HK. All authors have approved the final version of the manuscript.

## Conflict of Interest

The authors declare that the research was conducted in the absence of any commercial or financial relationships that could be construed as a potential conflict of interest.

## Publisher's Note

All claims expressed in this article are solely those of the authors and do not necessarily represent those of their affiliated organizations, or those of the publisher, the editors and the reviewers. Any product that may be evaluated in this article, or claim that may be made by its manufacturer, is not guaranteed or endorsed by the publisher.
